# Early and late indications of item-specific control in a Stroop mouse tracking study

**DOI:** 10.1371/journal.pone.0197278

**Published:** 2018-05-17

**Authors:** Carsten Bundt, Marit F. L. Ruitenberg, Elger L. Abrahamse, Wim Notebaert

**Affiliations:** 1 Department of Experimental Psychology, Ghent University, Ghent, Belgium; 2 School of Kinesiology, University of Michigan, Ann Arbor, MI United States of America; 3 Basque Center on Cognition, Brain and Language, San Sebastián, Spain; 4 IKERBASQUE, Basque Foundation for Science, Bilbao, Spain; Harvard University, UNITED STATES

## Abstract

Previous studies indicated that cognitive *conflict* continues to bias actions even after a movement has been initiated. The present paper examined whether cognitive *control* also biases actions after movement initiation. To this end, we had participants perform a Stroop task in which we manipulated the item-specific proportion of (in)congruent trials (80% congruent vs. 20% congruent). Importantly, participants responded via mouse movements, allowing us to evaluate various movement parameters: initiation times, movement times, and movement accuracy. Results showed that mouse movements were faster and more accurate during congruent trials compared to incongruent trials. Moreover, we observed that this congruency effect was larger for 80% congruent compared to 20% congruent items, which reflects item-specific cognitive control. Notably, when responses were initiated very fast – rendering virtually no time for stimulus processing before movement onset – this item-specific control was observed only in movement times. However, for relatively slow initiated responses, item specific control was observed both in initiation and in movement times. These findings demonstrate that item-specific cognitive control biases actions before and after movement initiation.

## Introduction

The ability to engage in goal-directed action is a core aspect of human cognition and has been referred to as cognitive control (e.g., [1]). In the laboratory, cognitive control can be investigated via conflict paradigms where stimulus features are potentially in conflict with each other. For example, in the Stroop task [2] participants respond to the ink color in which color words are printed. Responses are typically faster and more accurate for congruent (e.g., “GREEN” in green ink) compared to incongruent (e.g., “GREEN” in red ink) combinations of ink color and color word. It is assumed that the (semantic) processing of the task-irrelevant color word occurs in a relatively automatic fashion, such that it interferes with the processing of the task-relevant ink color and consequently impairs performance on incongruent trials (e.g., [3]). The difference in performance between responses to congruent versus incongruent stimuli is referred to as the *congruency effect* (e.g., [4]).

The congruency effect is not a static effect but is modulated, for example, by previous trial congruency. The so-called *congruency sequence effect* reflects the observation that when the previous trial was incongruent the congruency effect is typically decreased compared to when the previous trial was congruent [5]. The congruency sequence effect in the Stroop task is assumed to be the result of conflict-driven adjustments in cognitive control [4]. Another example, which is core to the current paper, is the impact of the overall proportion congruency within a specific trial list. Hence, the congruency effect is typically reduced for color words that are presented mostly with their incongruent color (mostly incongruent or MI items) as compared to color words that are presented mostly with a congruent color (mostly congruent or MC items). This phenomenon is referred to as the *item-specific proportion congruency (ISPC) effect* and represents a hallmark phenomenon of cognitive control [6–8].

The ISPC effect is often explained in terms of conflict monitoring and subsequent stimulus-driven attention modulation. More specifically, various studies have proposed that participants monitor conflict and modulate attention accordingly to maintain current task-goals [7, 9–14]. As a result, MC items become associated with a weak attentional filtering of the task-irrelevant stimulus feature (i.e., the word) over time, as this feature often does not interfere with the task-goal to correctly respond to the stimulus. In contrast, for MI items these models assume that the task-irrelevant stimulus feature becomes associated with a strong attentional filtering, as word information regularly interferes with the task-goal [6, 11, 13]. Alternatively, others have suggested that ISPC effects are due to the implicit acquisition of stimulus-response contingencies that are used to predict responses [15, 16]. However, an extensive discussion of both views is beyond the scope of the present paper.

So far, conflict processing and cognitive control in the Stroop task have typically been investigated via discrete responses (e.g., key presses or verbal responses). A limitation of such responses and their associated outcome measures (e.g., reaction time or accuracy) is that only the end product of action control – that is, the executed response – can be evaluated. To investigate conflict and cognitive control as it dynamically unfolds over time one can employ mouse tracking, as this exposes various action control components such as initiation and execution. To date, only a few studies have used this technique to study conflict processing and cognitive control. For example, Incera and McLennan [17] demonstrated that the Stroop effect could be observed in mouse movement trajectories. In addition, Scherbaum and colleagues [18] showed that mouse trajectories can also reflect the Simon effect, in which congruency between an irrelevant stimulus location and a response location benefits performance. Another study observed that the size congruity effect (i.e., better performance on a number-comparison task when numerical size and physical size are congruent) is reflected in mouse trajectories as well [19]. Notably, Scherbaum and colleagues [18] also reported that the congruency effect as seen in mouse trajectories was more pronounced when the previous trial was congruent, while the congruency effect was smaller when the previous trial was incongruent, indicative of a congruency sequence effect reflecting adjustments in cognitive control (c.f., [20]). These studies reinforce the notion that mouse tracking is suitable to examine simple conflict processing and cognitive control in conflict paradigms, yet they have revealed little information regarding the processing stage at which conflict and cognitive control set in.

Following the traditional viewpoint that information processing is composed of distinct stages that are serially organized and independent from each other [21], it could be hypothesized that effects of conflict processing and cognitive control will only be observed in parameters that reflect response selection processes preceding the onset of the actual action. However, recent studies have indicated that cognitive conflict may bias actions both before and after movement initiation [22–24]. Using a continuous response measure, Buc Calderon et al. [22] had participants perform a reaching task in which lateral targets were (in)correctly cued. They manipulated the probability of the cue validity such that one type of cue indicated a high probability of cue-target congruency, while another type indicated a low probability. Results showed that reach trajectories were biased by the probability manipulation, which suggests an ongoing competition between response alternatives even after action planning and during the action execution phase. In another study by Cohen Kadosh et al. [24], functional magnetic resonance imaging (fMRI) was used to evaluate the neural correlates of cognitive conflict as reflected in the size-congruity effect. Results indicated that the congruency effect was reflected in activation of the primary motor cortex during response performance, suggesting that cognitive conflict continues to affect actions even after movement initiation.

Taken together, mouse tracking has mainly been employed to examine conflict processing [17–19] and (to a lesser degree) congruency sequence effects [18] in conflict tasks. The effect of conflict processing and congruency sequence effects on mouse trajectories may be explainable by modern theories on cognitive control. These theories (e.g., [25, 26]) argue that binding between task-relevant cortical areas and stimulus features is facilitated in situations where conflict is experienced, and that conflict affects performance because network weights are adjusted via Hebbian (associative) learning after every trial. From this perspective, however, it remains unclear how conflict adaptation unfolds within-trial and how such changes in within-trial cognitive control dynamically affect responses. Therefore, examining how item-specific control affects mouse trajectories is especially relevant as it allows to investigate how rapid, within-trial conflict adaptation biases response selection at different moments in time.

The present study therefore aimed, first, to evaluate whether item-specific control can be observed in mouse movements and, second, to examine if item-specific control biases mouse responses until and/or after response initiation. To that end, participants were presented with four response boxes with a color word printed in them in the upper half of a computer screen. After they clicked on a start button that was shown centrally in the lower part of the computer screen, a Stroop stimulus was displayed. Participants were instructed to respond to the ink color of this stimulus by moving the mouse cursor towards one of the four response boxes that corresponded with the ink color of the Stroop stimulus. Importantly, we manipulated the proportion of (in)congruent stimuli in an item-specific manner, such that some words were in a congruent word-color combination on 80% of the trials (i.e., MC items), while others were presented in such a congruent combination on only 20% of the trials (i.e., MI items). We recorded the initiation time (IT), movement time (MT), and movement accuracy in terms of deviation from the optimal trajectory (area under the curve or AUC) of each of the participants’ mouse movement responses. We hypothesized mouse tracking to be able to capture item-specific control processes. Furthermore, while traditional (sequential) information processing accounts [21] would predict that item-specific control only affects ITs as response selection would precede action execution, more recent accounts argue that response selection continues after movement initiation [22–24]. In line with these recent accounts, we predict that response selective processes will continue after movement initiation, such that the ISPC manipulation will be evident after response initiation in mouse movements as well.

## Method

### Participants

Thirty-one psychology students of Ghent University participated in the experiment. The data from one participant (who reported to be dyslexic) were removed, and as such the analyses reported below are based on the data of 30 participants (7 male; 5 left-handed). They were aged between 17 and 25 years (*M* = 18.87, *SD* = 1.66) and were native Dutch speakers. None of the participants reported to have problems with their sight (e.g., color blindness; corrections via glasses or contact lenses were allowed). Prior to the experiment, all participants provided written informed consent (please note that given the situation in Belgium that it is not unusual for 17-year-old individuals to attend university, consent by the legal guardian is not required for such basic cognitive research). They received partial course credits for their participation. The study was approved by the ethical committee of the Faculty of Psychology and Educational Sciences of Ghent University.

### Apparatus

Stimulus presentation, timing, and data recording were controlled by MouseTracker software [27], running on a Dell Latitude E5530 laptop computer. The laptop was connected to a 48cm Dell monitor, on which the stimuli and response locations were presented. Participants used a standard computer mouse with the cursor speed set at the 6/11 default mode in Windows 7.

### Task and procedure

**Fig 1:**
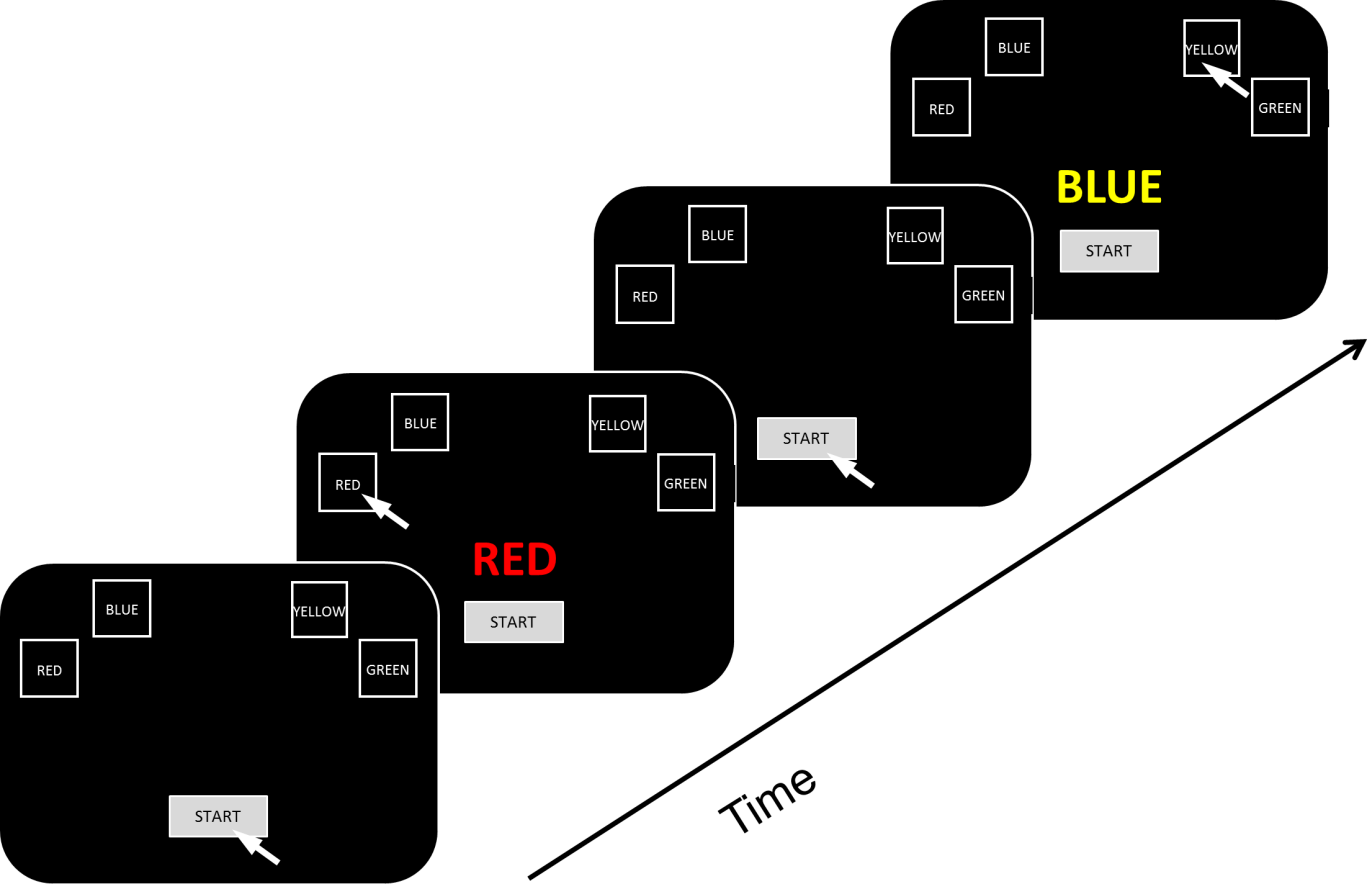
Schematic illustration of a congruent (“RED” in red ink) and an incongruent trial (“BLUE” in yellow ink) trial. Participants clicked the start button to prompt the presentation of a stimulus that indicated the correct response box. Note that all words were actually displayed in Dutch and that there was a 300ms delay between clicking the start button and stimulus presentation. In addition, after clicking the response box of their choice, participants had to wait 500ms before the start button appeared again to start the next trial.

Before the start of the experiment, participants were briefly informed about the procedure and provided written informed consent. They then completed a short questionnaire regarding their demographic details and filled out Annett’s [28] Handedness Inventory. Next, they were seated in front of a computer screen. As Fig 1 shows, participants were presented four black response boxes (4cm×5cm) outlined in white against a black background. The boxes were displayed at the top of the screen and were filled with a color-word that was printed in white letters (from left to right “ROOD”, “BLAUW”, “GEEL”, and “GROEN”; the Dutch words for “RED”, “BLUE”, “YELLOW”, and “GREEN”, respectively). The response boxes remained visible throughout the experiment.

The stimuli in the present experiment consisted of four Dutch color-words for red, blue, yellow and green. These words were printed in one of the same four ink colors, with the restriction that red and green were always printed in either red or green, and blue and yellow always in either blue or yellow. These color-words and ink colors were paired so that first contingency problems were avoided [29]. Specifically, in a canonical four-alternative forced-choice task, increasing the proportion of congruent stimuli (e.g., 50% “RED” in red ink color) would result in the irrelevant dimension (i.e., the word) becoming predictive (and therefore relevant) of the correct response over time if the number of task irrelevant features (i.e. possible ink colors: blue, yellow, green) was not reduced accordingly [30]. Second, color-words and ink colors were paired in this specific way to optimize mouse tracking (i.e., for incongruent trials, the correct response and conflicting response were always on opposite sides from the start location). For one of the pairs, 80% of the trials were congruent, while for the other pair only 20% of the trials were congruent (counterbalanced across participants). This allowed examination of the ISPC effect.

At the beginning of each trial, a start button (2cm×5cm) appeared centrally at the bottom of the display. Upon clicking the button, the button and mouse cursor disappeared. After a 300ms interval (c.f., [31, 32]) a stimulus was presented just above the start button location and the mouse cursor reappeared. The interval was included to separate the motor component of clicking the start button from the motor component of initiating the mouse movement, and to allow participants to shift their attention from the start click to the stimulus location. After clicking the start button, the cursor was automatically relocated to the center of the start button (*x*,*y* coordinates 0,0.16), so that mouse trajectories always started from the same location. Participants were instructed to move the mouse as quickly as possible from the start button to the response box of which the color-word correctly corresponded with the ink color of the stimulus and click the box to register their choice. The distance between the start location of the mouse cursor and the center of each response box was approximately 31.5cm. Participants could use their preferred hand for responding. A trial ended once they clicked one of the response boxes. When participants clicked the wrong box, a red ‘X’ appeared centrally on the display for 2s, after which they could start the next trial. The inter trial interval was 500ms, meaning that following their response participants had to wait 500ms before the start button appeared again to start the next trial. When participants initiated the mouse movement later than 500ms after stimulus presentation, the trial was followed by a message encouraging them to start moving earlier on subsequent trials. This was done to ensure that participants did not strategically wait to initiate their movement until after selection of the correct response was fully completed (c.f., [19, 33]).

Participants first performed a brief practice block in which they performed 16 randomly ordered trials (50% congruent). Next, they completed four experimental blocks and could take a self-paced break after each block. In two of the blocks, the ink color of the Stroop stimuli followed a fixed order. Consequently, responses also followed a fixed order. Each of these *sequence blocks* involved 20 repetitions of an 8-element sequence that was constructed such that it did not involve direct repetitions, reversals or runs of three. The sequence was always a version of the form 13243142, and counterbalanced across response locations and participants. In the other two blocks, the Stroop stimuli were randomly ordered. These *random blocks* involved the presentation of 160 trials, with the restrictions that there was no direct repetition of the stimulus color (and thus response location) and that each stimulus was presented equally often. The sequence and random blocks alternated and the order was counterbalanced across participants. Initially, we aimed to examine whether the predictability of response order modulates the ISPC effect. Participants showed faster mouse movements in sequence than in random blocks (1196 vs. 1220ms), *F*(1,29) = 7.51, *p* = .010, *η*_*p*_^*2*^ = .21, and mouse trajectories were more accurate in sequence than in random blocks (.45 vs. .48), *F*(1,29) = 4.39, *p* = .045, *η*_*p*_^*2*^ = .13. However, results showed no indications that response order significantly affected the ISPC effect in any of our dependent variables (*ps*>.545). Therefore, for the sake of simplicity, we decided to omit the factor response order from the analyses below and do not further discuss the sequence manipulation.

### Data processing

The software recorded the *x-* and *y*-coordinates of the mouse cursor every 13-16ms during each trial (±70Hz sampling rate). In addition, it recorded performance time during each trial by logging how many milliseconds elapsed since stimulus presentation. The raw trajectory data were rescaled to standardized coordinate space (*x*-axis range -1 to 1; *y*-axis range 0-1.5) and remapped toward the right. Trajectories were time-normalized by the software to 101 time-steps, allowing us to average across the full length of trials that varied in duration (for more details on the rescaling and normalization procedures, see [27]).

Based on these data we determined IT, MT, and AUC to investigate participants’ performance. IT refers to time in ms between stimulus presentation and onset of the mouse movement (i.e., the moment that the *x*,*y* coordinates first differ from those at the start position). MT is the total time in ms the participant needed to move the mouse from the start button to click on the response box. The AUC is the geometric area between the recorded mouse movement and the optimal trajectory (which is a straight line from the start button to the response box). This measure is based on the normalized trajectory data and is thus dimensionless. It reflects the overall attraction towards a response alternative – a greater area indicates stronger attraction to the alternative and more conflict.

We calculated each of these measures (i.e. IT, MT, AUC) for congruent and incongruent trials within the 80% congruent pairs and 20% congruent pairs for every participant. The first trial of each block, trials on which an error was made (<1%) and trials following an error were omitted from the analyses. In addition, trials were excluded from the analyses when the MT exceeded more than 3 standard deviations from the mean across participants. This was done separately for the congruent and incongruent trials within the two proportion congruent conditions, and resulted in the removal of 1.7% of the trials.

## Results

We performed repeated measures analyses of variance (ANOVAs) on each of our dependent variables with Congruency (2; congruent vs. incongruent) and Proportion Congruency (2; 80% congruent vs. 20% congruent) as within-subject variables. The results of these ANOVAs are shown in Table 1. Results of our ANOVA on MT showed that mouse movements were performed slower for incongruent than for congruent trials (1275 vs. 1142ms), *F*(1,29) = 106.31, *p*<.001, *η*_*p*_^2^ = .79. This congruency effect was also observed in the accuracy of mouse movement trajectories, as results showed that AUC was larger for incongruent relative to congruent trials (.57 vs. .35), *F*(1,29) = 55.27, *p*<.001, *η*_*p*_^2^ = .66. Moreover, IT tended to be shorter for congruent compared to incongruent trials (207 vs. 212 ms) but this difference just failed to reach significance, *F*(1,29) = 3.94, *p* = .057, *η*_*p*_^2^ = .12. Results further showed a main effect of Proportion congruency on AUC. Specifically, AUC was larger for MC relative to MI items (.49 vs. .43), *F*(1,29) = 4.50, *p* = .043, *η*_*p*_^2^ = .13). This main effect was not significant for IT (210 vs. 208 ms, *F*<1), and MT (1210 vs. 1196 ms, *p* = .25).

**Table 1:** Overview of the main and interaction effects of the ANOVAs with congruency (2) and proportion congruency (2) on IT, MT, and AUC for the main analyses and the IT bin analyses.

	Main ANOVA			IT_fast_			IT_slow_		
	F	p	ηp2	F	p	ηp2	F	p	ηp2
**IT**									
Congruency	3.94	.057	.12	.83	.37	.03	6.13	.019	.175
Proportion congruency	.71	.41	.02	.89	.88	.00	.06	.81	.00
ISPC	2.37	.14	.08	.00	.96	.00	11.37	.002	.28
**MT**									
Congruency	106.31	<.001	.79	90.78	<.001	.76	100.68	<.001	.78
Proportion congruency	1.40	.25	.05	1.44	.24	.05	1.44	.24	.05
ISPC	53.76	<.001	.65	14.11	.001	.33	46.12	<.001	.53
**AUC**									
Congruency	55.27	<.001	.66	50.37	<.001	.64	41.94	<.001	.59
Proportion congruency	4.5	.043	.13	2.79	.11	.09	6.21	.019	.18
ISPC	15.35	<.001	.35	3.28	.081	.10	20.68	<.001	.42

**Fig 2:**
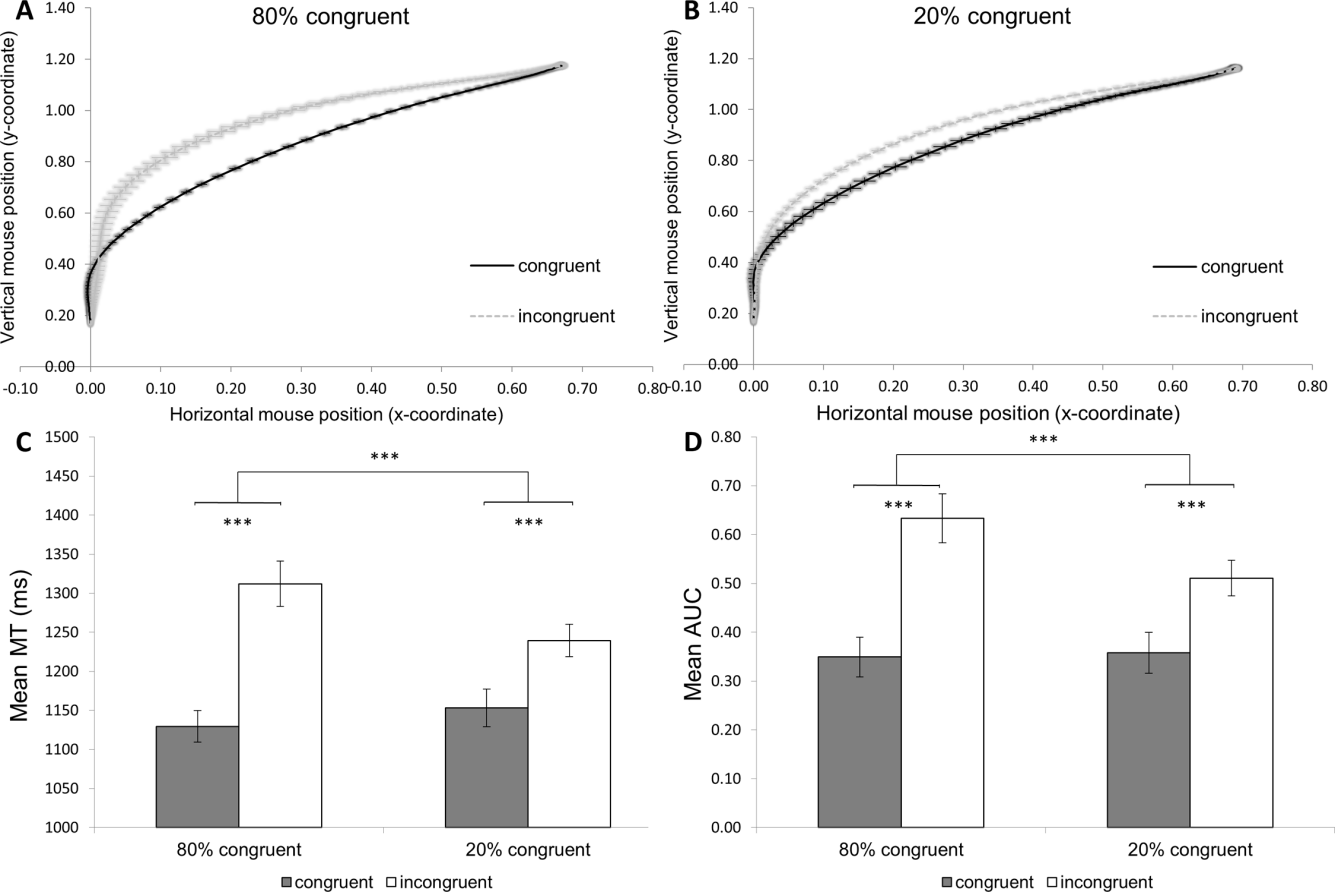
Panel A and B: Mean mouse trajectories for the congruent and incongruent trials as a function of proportion congruency (80% congruent: Panel A; 20% congruent: panel B). Error bars represent 95% confidence intervals. Panel C and D: Mean MT and AUC for the congruent and incongruent trials as a function of proportion congruency (i.e., ISPC effect). Error bars represent standard errors. ***p<.001.

Most interestingly, our results revealed significant Proportion Congruency × Congruency interactions for MT and AUC indicative of the ISPC effect (see Table 1). Fig 2 shows the mouse movement trajectories for congruent and incongruent trials in the 80% congruent (panel A) and 20% congruent (panel B) items. In addition, Fig 2 shows participants’ performance as reflected in MT (panel C) and AUC (panel D) for the congruent and incongruent trials as a function of proportion congruency. In all figures, error bars showing standard errors were calculated via the Cousineau-Morey method for repeated-measures and represent within-subject standard errors [34, 35]. Here we first zoom in on the interaction for MT, *F*(1,29) = 53.76, *p*<.001, *η*_*p*_^2^ = .65. Participants showed shorter MTs on congruent compared to incongruent trials for MC items (1129 vs. 1311ms), *F*(1,29) = 122.54, *p*<.001, *η*_*p*_^2^ = .81 , as well as for MI items (1155 vs. 1239ms), *F*(1,29) = 46.09, *p*<.001, *η*_*p*_^2^ = .61. A paired sample *t*-test revealed that the congruency effect was larger for MC compared to MI items (182 vs. 84 ms), *t*(29) = 7.33, *p*< .001, demonstrating the ISPC effect. Results of the ANOVA on AUC also showed an ISPC effect, *F*(1,29) = 15.35, *p*<.001, *η*_*p*_^2^ = .35. Participants’ mouse movements exhibited a larger AUC on incongruent relative to congruent trials, for both MC items (.64 vs. .35), *F*(1,29) = 57.20, *p*<.001, *η*_*p*_^2^ = .66, and MI items (.51 vs. .36), *F*(1,29) = 25.60, *p*<.001, *η*_*p*_^2^ = .48. Like with MT, paired *t*-tests indicated that this congruency effect was larger for MC compared to MI items (.29 vs. .15), *t*(29) = 3.92, *p*<.001. Results of the ANOVA on IT did not reveal an ISPC effect, *F*(1,29) = 2.37, *p* = .14, *η*_*p*_^2^ = .08.

To rule out that the congruency and the ISPC effects varied as a function of response location (peripheral vs. central pair), we performed two additional analyses. First, we ran an ANOVA with Location and Congruency as within-subject variables. Results demonstrated that Location did not significantly interact with the congruency effect for any of our dependent variables (*p*s>.64). Second, we ran an ANOVA with Proportion Congruency and Congruency as within-subject variables, and with Location (peripheral MC vs. peripheral MI) as between-subject variable. Results showed that the Congruency main effect as well as the Proportion Congruency × Congruency interaction remained significant for both MT and AUC, *F*s>14.84, *p*s< = .001, but that Location did not interact with these effects for any of our dependent variables (*p*s>.50).

Inspection of our data indicated that some trials were associated with extremely fast ITs (*M =* 208 ms, *SD* = 171 ms). It is reasonable to assume that on very fast initiations, stimuli were not fully processed before the initiation. Therefore, it can be expected that substantial differences exist in conflict processing between trials with relatively short versus trials with relatively long ITs. To examine this possibility, we ranked all trials based on IT and divided them based on a median split into two bins. This was done separately for the congruent and incongruent trials and for each participant, and resulted in a fast bin (IT_fast_, *M* = 88.30 ms, *SD* = 55.47) and a slow bin (IT_slow_, *M* = 330.27 ms, *SD* = 101.1). We then performed a repeated-measures ANOVAs on IT, MT, and AUC with Bin (2; IT_fast_ vs. IT_slow_) added as a within-subject variable.

**Fig 3:**
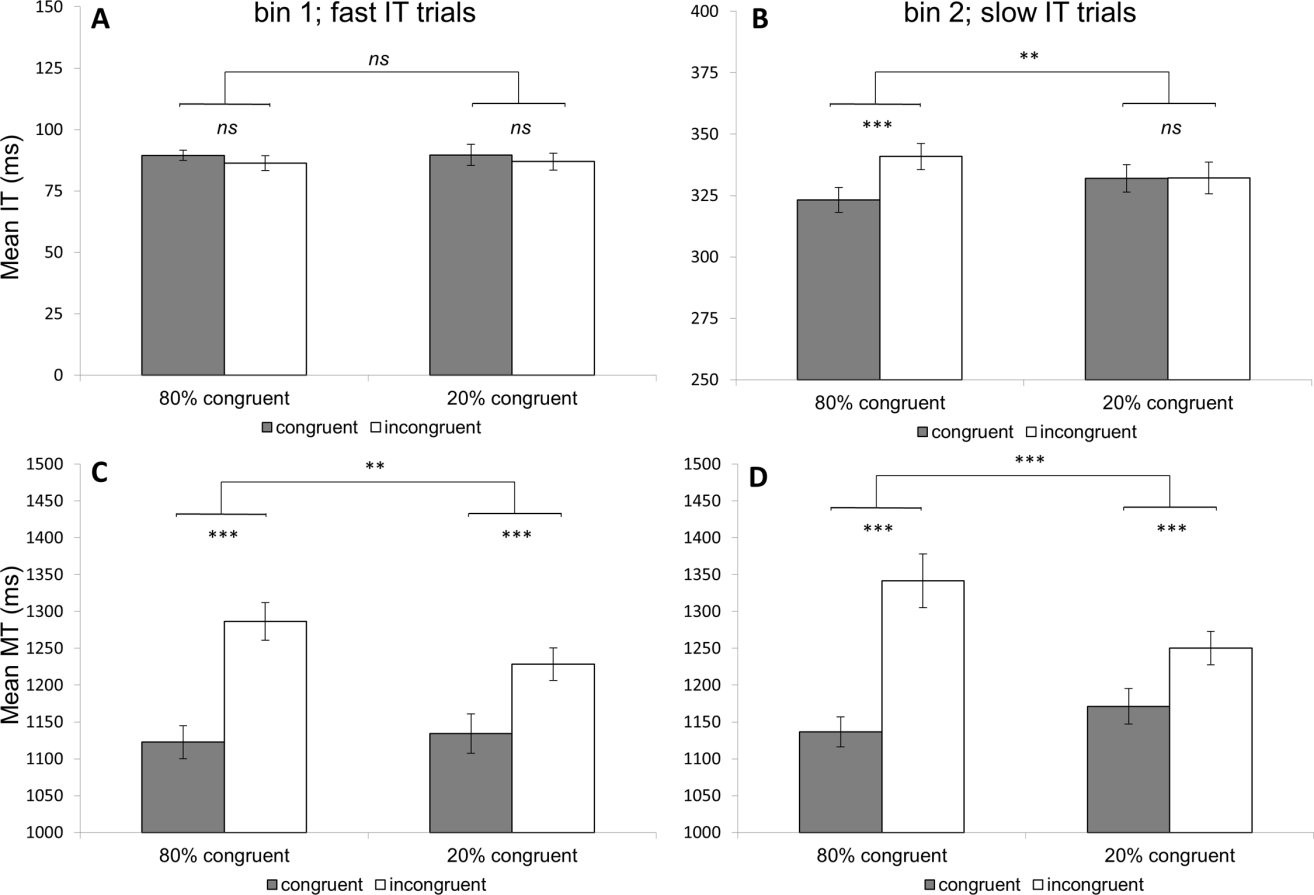
Mean IT (panel A and B) and MT (panel C and D) for fast and slow initiation time bins. Error bars represent standard errors. **p<.01, ***p<.001.

The ANOVAs revealed a significant (three-way) interaction between the ISPC and bin for IT, *F*(1,29) = 10.14, *p* = .003, *η*_*p*_^2^ = .26, and MT, *F*(1,29) = 6.29, *p* = .018, *η*_*p*_^2^ = .18. As Table 1 shows, the ISPC effect was significant for the slow IT trials (see Fig 3B), while results showed no ISPC effect for fast IT trials (see Fig 3A). For MT, the ISPC effect was observed in both fast IT and slow IT trials (see Fig 3C and 3D, respectively), although a paired-samples *t-*test showed that the ISPC effect was smaller for fast IT compared to slow IT trials (67 vs. 131 ms), *t*(29) = -2.51, *p* = .018. For AUC, results showed that trajectories were less accurate for fast IT compared to slow IT trials (.50 vs. .42), *F*(1,29) = 9.84, *p*<.001, *η*_*p*_^2^ = .41. Results further confirmed that there was a general ISPC effect, *F*(1,29) = 14.85, *p* = .001, *η*_*p*_^2^ = .34, but this effect was not modulated by Bin (*p* = .14).

## Discussion

The present study examined whether item-specific control biases actions even after the initiation of the corresponding movement. We employed a Stroop task in which we manipulated the item-specific proportion congruency while participants’ responses were recorded using mouse tracking. We showed for the first time that the ISPC effect can be observed in various mouse tracking parameters. More importantly, our results revealed that item-specific control affected response selection after movement onset – even when stimuli were (at least partially) processed during the interval between stimulus presentation and movement onset. This is an interesting observation because traditionally stimulus identification, response selection, response initiation, and response execution are viewed as distinct, serial information processing stages that neither overlap nor interact [21]. According to this view, response selection is assumed to be completed at the time of response initiation and any processes after this point are thought to be unaffected by prior factors (e.g., cognitive conflict and cognitive control). Our study is in line with a set of recent studies that already casted doubts on the existence of a strictly serial information processing system. For example, these studies demonstrated at the behavioral [22], neurophysiological [23], and neural level [24] that cognitive conflict biases actions even after the response initiation phase. Our study extends these findings as the current results suggest that information processing both before and after initiation of a response was under the influence of item-specific control. Even when trials were initiated slowly enough such that stimulus processing could occur before response initiation, our ISPC manipulation was still evident in mouse movement execution, thus reflecting adaptive, item-specific response selection processes occurring after movement initiation. The notion that cognition biases actions after movement initiation is further supported by our finding that compared to relatively slow initiated mouse movements, relatively fast initiated movements deviated more from the optimal trajectory. This suggests that participants experienced a stronger attraction to the alternative response when there was little time to process a stimulus before movement initiation.

In addition to the here observed implementation of item-specific control (rather than simple conflict) both before and after movement onset, the current study extends previous work in another way. Most of the previous studies showing that cognition leaks into the response execution interval involved intentional, top-down processes based on the learning and implementation of instructed rules. Conversely, in the present study we demonstrate that incidental, bottom-up control processes that are triggered within the trial (i.e., in an ISPC task, only upon stimulus presentation does the system know the required control demands for the ongoing trial) can also bias responses both before and after movement onset. This differs greatly from, for example, the explicit cues used by Buc Calderon et al. [22] to indicate the probability of an upcoming stimulus. By demonstrating that bottom-up, item-specific control affects mouse trajectories after movement onset, we provide novel insights into the circumstances under which cognitive processing and response execution can temporally overlap.

Our finding that the typical congruency effect was reflected in mouse movements corroborates previously reported observations [17–19]. Consistent with an ISPC effect, our results showed that the congruency effect was smaller for MI items compared to MC items in mouse movement parameters MT and AUC. These observations are in line with previous research demonstrating that performance in key-press tasks is modulated by the item-specific proportion of (in)congruent trials [6–8], potentially due to differential allocation of attentional resources to the task-irrelevant stimulus feature in the MI compared to the MC context [7, 36]. The present results extend these findings by showing that mouse movement responses are able to capture the influence of item-specific control. The observation that the difference in mouse movement deviations from the optimal trajectory between incongruent and congruent items was enhanced for MC compared to MI items suggests that participants experienced a stronger attraction to the response associated with the task-irrelevant stimulus feature on incongruent trials for MC items. This may underlie the modulation of movement times by item-specific control.

It may appear counter-intuitive that our data showed a smaller ISPC effect in MT for trials in the IT_fast_ compared to IT_slow_ bin, because the demand for the implementation of control processes may be strongest closer to target stimulus onset. However, Pratte and colleagues [37] have shown that indices of cognitive control (such as congruency) in the Stroop task are smallest for fast responses and increase with increasing response latency. In light of these findings, the smaller ISPC effect in MT for trials in the IT_fast_ compared to IT_slow_ bin in the current study may be due to the fact that only little time elapsed between stimulus presentation and the initiation of movements, leaving insufficient time for an ISPC effect to unfold completely. Alternatively, the smaller ISPC effect in MT for trials in the IT_fast_ compared to the IT_slow_ bin may also be related to stimulus and response anticipation and repetitions that may have prohibited the ISPC manipulation to have a rapid effect on movement initiation. Admittedly, future research needs to verify these speculations.

In the current study, participants were required to identify the color of a centrally presented Stroop stimulus in order to then move the mouse cursor towards the response box that included the corresponding written color word. One limitation of the experimental design may be that response alternatives were presented as color words. To identify the correct target, participants had to activate (at least to some extent) their semantic processing system, while this system ideally should be suppressed when processing Stroop stimuli to minimize the impact of the task-irrelevant stimulus feature. It could therefore be argued that the concurrent need for both inhibition and activation of the semantic processing system may have influenced participants’ performance in the current task. An alternative approach might have been to label the response boxes by color patches such that participants had to simply visually match the ink color of the Stroop stimuli with the color of the response boxes. It has been shown that responses are faster when one needs to discriminate between items that share modality (e.g., matching one color with another color) compared to when items differed in their modality (e.g., matching color with color word semantics) [38]. Accordingly, generally (much) faster response times may have been the result if response boxes would have been labeled by ink color in the current task. However, when responses become faster the Stroop effect may be on the line as the effect is known to decrease with shorter reaction times [37]. We argue that if response boxes were labeled by ink color reaction times would decrease, which would result in either a reduction or even a cancellation of congruency effects (i.e., our effects of interest). Future studies should examine the impact of the match between stimulus and response modality on the congruency and ISPC effects as reflected in mouse movement parameters.

Another limitation of our paradigm is that we cannot exclude the possibility that our data may reflect two different types of trials: one trial type in which individuals selected the correct action before movement onset and a second trial type in which individuals initially selected an incorrect action, which they then need to correct in order to move towards the correct target. Even if this was the case, this is still in line with the notion of interacting rather than sequential processing stages during action selection, as we speculate that the correction of an initially erroneous action selection may be corrected (even) after movement initiation and is not dependent on the resolution of such a selection error before movement onset. Our speculation is in line with recent accounts that have proposed that behavior is governed by a constant and parallel competition and implementation of multiple action alternatives [39]. According to this view, evidence for each action alternative is accrued continuously and throughout the movement itself. Correspondingly, if evidence is accrued in favor of the incorrect response alternative at some moment during the movement, such selection error can subsequently be adjusted by accruing evidence in favor of the selection of the correct response. To that effect, the correction of such erroneous selection does not require the process of response selection to begin anew, thus arguing in favor of continuous response selection both before and after movement onset.

In conclusion, the present study illustrates that item-specific control affects mouse movement responses before and also after movement onset. In line with recent evidence, our findings support the notion that response selection may not necessarily be completed before movement initiation, but that cognitive processes continue to affect responses even after movement initiation. These findings illustrate the potential of mouse tracking to study cognitive control. In addition to capturing simple congruency effects [17, 19], this technique is also suitable for assessing the action dynamics of cognitive control phenomena such as the congruency sequence effect [18, 20] and the ISPC effect (present Stroop task). Future studies should examine the potential of mouse tracking to evaluate the action dynamics of ISPC effects and other measures of cognitive control in different paradigms, taking into account any effects of stimulus-response modality compatibility (c.f., [38]).
